# Mitigating Evidentiary Bias in Planning and Policy-Making: Comment on "Reflective Practice: How the World Bank Explored Its Own Biases?"

**DOI:** 10.15171/ijhpm.2016.96

**Published:** 2016-07-20

**Authors:** Justin Parkhurst

**Affiliations:** London School of Hygiene and Tropical Medicine, London, UK.

**Keywords:** Evidence and Policy, Evidentiary Bias, Cognitive Bias, Policy-Making Institutions

## Abstract

The field of cognitive psychology has increasingly provided scientific insights to explore how humans are subject to unconscious sources of evidentiary bias, leading to errors that can affect judgement and decision-making. Increasingly these insights are being applied outside the realm of individual decision-making to the collective arena of policy-making as well. A recent editorial in this journal has particularly lauded the work of the World Bank for undertaking an open and critical reflection on sources of unconscious bias in its own expert staff that could undermine achievement of its key goals. The World Bank case indeed serves as a remarkable case of a global policy-making agency making its own critical reflections transparent for all to see. Yet the recognition that humans are prone to cognitive errors has been known for centuries, and the scientific exploration of such biases provided by cognitive psychology is now well-established. What still remains to be developed, however, is a widespread body of work that can inform efforts to institutionalise strategies to mitigate the multiple sources and forms of evidentiary bias arising within administrative and policy-making environments. Addressing this gap will require a programme of conceptual and empirical work that supports robust development and evaluation of institutional bias mitigation strategies. The cognitive sciences provides a scientific basis on which to proceed, but a critical priority will now be the application of that science to improve policy-making within those agencies taking responsibility for social welfare and development programmes.

The World Bank’s recognition of how cognitive biases can lead to errors by its own expert staff (see editorial by Mckee and Stuckler)^1^ can be interpreted in two ways. It can be seen as an important example of transparent critical self-reflection by powerful global institution, or it can be seen as a long-overdue step to act upon a well-established scientific field with implications for policy and practice. It is likely an example of both.

McKee and Stuckler call for more institutions to adopt ‘reflective practice’ mirroring that seen in the World Bank’s 2015 World Development Report.^2^ Yet recognition of the need for critical self-reflection is not new, being called for by Mannheim, for instance, in his early work outlining the sociology of knowledge (first published in English in 1936).^3^ However, it is indeed rare for global policy-influencing bodies to undertake such reflections so openly, especially when it can equate to admission of past errors. The World Bank has not been without controversy, at the centre of long running debates about whether particular development approaches (eg, structural adjustment programmes) have caused more harm than good in low-income settings.^4^ As such the organisation could be particularly sensitive to showing that its experts are subject to bias. The agency must also justify its budget to its donor governments, which can further incentivise the non-admission of faults. In such ways, it is refreshing to see such an open reflection on how error-prone the agency can be, which can indeed serve as an example for many other organisations as McKee and Stuckler’s express.

But the fact that humans are prone to evidentiary bias and judgement errors (what has often been termed ‘irrationality’) arising through cognitive processes, is something that has been recognised historically. We can, therefore, equally question why we have not done more to address this in our policy-making institutions – settings in which evidentiary errors can have profound implications for decisions affecting human life and welfare. Mannheim himself refers even further back to Francis Bacon’s 17th century *Novum Organum Scientarium* [New Instrument of Science], which identified a set of ‘idols’ that (as described by Zargorin) capture “the mental, psychological and socially engendered dispositions and beliefs that [are] responsible for systematic distortion and error (pp. 387).”^5^

Indeed, some of the World Bank’s own errors have been well-known for some time. A widely-cited reading in the field of international development, for instance, is Ferguson’s 1994 book *The Anti-Politics Machine*,^6^ which, two decades ago, illustrated how the World Bank interpreted evidence about Lesotho in repeatedly biased ways. The World Bank, to its credit, acknowledges Ferguson’s insights in the 2015 World Development Report. Yet it is clear that there is a need for organisations involved in social policy development to better integrate the learning and knowledge we have about cognitive bias into their institutional systems and structures to prevent such errors from occurring, or to recognise them more quickly when they do occur.

While the existence of unconscious bias has been recognised for centuries, what the field of cognitive psychology has particularly brought to this topic is a scientific rigour to study the origins and mechanisms by which they arise. What has arguably been lacking, then, is greater application of these insights within policy-making systems. Indeed, just such a gap was identified by the US National Research Council in 2012, which concluded that while:

“[t]here is an extensive literature in cognitive social psychology and behavioral decision theory on how people make judgments, decisions, and choices…. These sciences have not…been applied to collective reasoning and group decision-making *in public policy settings* at anything close to the level needed” (pp. 57, emphasis in original).^7^

But what would it mean to address this gap, and to apply these insights into such settings in a more systematic, and scientifically grounded manner?

McKee and Stuckler end their editorial pointing to possible remedies. Some of these are individually oriented – like increasing awareness of the problem – while others are institutional, such as removing incentives that lead to particular biases, or formalising rules that force decision-makers to see issues from a different perspective. This kind of thinking should be seen as a critical next step in the application of the cognitive sciences to improve decision-making, but current efforts serve as only the tip of a much broader field of future work. Specifically, a great deal more remains to be done to understand both how decision-making biases arise, as well as how to institutionalise structures, rules, and practices that make biased uses of evidence less likely, more obvious, and/or more manageable. Institutional changes will need to be based on both conceptual insights and empirical validation if they are to provide sustained and successful responses to these challenges.

In clinical medicine, some initial thinking in this area is taking place. The use of checklists to avoid certain diagnostic or treatment errors has been increasingly established^8^ – although it has been noted that there have not yet been extensive evaluations of this approach.^9^ Yet while basic errors may arise from so-called fast thinking in time-critical setting (which checklists often address), Seisha et al^10^ have used the term ‘cognitive biases plus’ to conceptualise other drivers of bias such as ‘groupthink,’ herd effects, or misaligned incentives that occur at organisational levels and undermine the practice of evidence-based medicine. Croskerry has similarly identified over 30 cognitive sources of diagnostic error, and 10 basic strategies for ‘cognitive debiasing’ to help avoid errors. ‘Metacognition’ is held up as a central approach – capturing the process of stepping back from decisions to critically reflect on potential bias.^11^ In a later article, Croskerry et al further identified a large number of ‘forcing functions’ which would mandate a step to be taken by a clinician in a decision process^12^; steps that could be institutionalised, for instance, by establishing formal rules about when checks or confirmation steps must happen before progression in treatment.

Some of these ideas are echoed in the critical reflection seen by the World Bank, but it is important to recognise there can be important differences between overcoming bias in clinical decision-making and within social policy decision processes. Policy decisions are rarely simple technical exercises aiming to achieve a universally agreed goal. Rather, policy-making typically involves making decidedly political choices over which course of action to follow, involving multiple competing priorities and potential disagreement about relevant outcomes to pursue. This reality, however, means that cognitive bias may arise in a number of different ways in policy processes. In a forthcoming work, I attempt to develop a ‘cognitive-political framework’ that maps out how key features of policy problems such as their contestation, complexity or uncertainty, and polarisation can manifest in different forms of evidentiary bias through different mechanisms.^13^ So, for instance, while numerical errors may arise from ‘fast’ intuitive thinking in complicated situations, in highly polarised political debates, there can be different pressure towards what has been termed ‘identity protective cognition’ in which individuals interpret evidence in ways that align with the values of their existing affinity group.^14^ Indeed, studies have even showing that more numerate or more cognitively able individuals are *more* likely to interpret evidence in biased ways in polarised or highly contested political debates.^15^ Depending on the origins of bias, then, different mitigation strategies will likely be required.

There is still much to learn, however, to construct strategies for debiasing policy decision-making processes. Progress will require both conceptual and empirical work to inform the institutionalisation of strategies that can address forms of bias specific to the decision-making environment. Institutionalisation of such efforts is particularly important for several reasons. First, staff turnover and strategic shifts mean that critical awareness training can be limited in duration of effect. Changing institutional rules and processes, or shifting how incentives influence the decision points in the first place, may prove more sustainable as individuals come and go from institutional environments. Second, not all sources of bias arise from individual errors, with decision spaces promoting polarisation or group-think generating particular forms of bias outside individual control in some contexts. Organisational arrangements may, thus, also be important targets for bias reduction strategies – for example, through efforts aiming to construct deliberative spaces that facilitate listening and learning across divided groups^16,17^ or which reduce so-called enclave deliberation.^18^ And finally, given that humans are naturally prone to bias, resultant errors will be a continual and ongoing problem that systems need to be structured to address from the start, not one off problems to solve at a single points in time.

At the moment, institutional bias-mitigation strategies in policy-making environments are often reported anecdotally, or not explicitly evaluated. So while it is known that misaligned incentives can facilitate bias, and while there have been calls to build political spaces to overcome polarisation (which evidence has shown can drive bias), there is not yet a strong evidence base testing whether, in practice, particular strategies reduce specific types of cognitive bias and evidentiary errors. Indeed, even limited to the more specific case of clinical diagnostic errors arising from heuristic shortcuts, a recent systematic review found only a handful of tested interventions, showing heterogeneous results so far.^19^

Taking the next steps in this area will require building a field of conceptual and applied science to more systematically develop and test bias-reducing interventions in institutional settings. We are likely only just beginning to see the use of interventions of this kind, but the cognitive sciences provide a body of work on which to build plausible hypotheses on how to mitigate bias in policy-making or planning environments. Future work will require rigorous evaluation designs to test the efficacy of bias mitigation strategies, as well as critical thinking on the generalisability of efforts shown to produce effects in particular decision-making environments.

Historically, we have not had a lack of recognition of our inherent tendencies towards evidentiary bias. Rather, we have seen a lack of integration of that knowledge into how we plan and act within those decision-making bodies making critical choices affecting human welfare. Humans may indeed be ‘irrational,’ but as Airely has noted, they are ‘predictably irrational.’^20^ What is needed now is to develop the scientific basis of how social policy-making institutions can integrate this knowledge to best avoid, identify, or mitigate cognitive biases and their associated errors.

## Acknowledgements

Justin Parkhurst is supported by a grant from the European Research Council [GRIP-Health: Getting Research into Policy in Health, grant number #282118].

## Ethical issues

Not applicable.

## Competing interests

Author declares that he has no competing interests.

## Author’s contribution

JP is the single author of the paper.
